# Recent advances in the systemic treatment of gastrointestinal stromal tumors

**DOI:** 10.20892/j.issn.2095-3941.2023.0302

**Published:** 2023-10-20

**Authors:** Robin L. Jones, Marin Golčić

**Affiliations:** 1 Sarcoma Unit, The Royal Marsden NHS Foundation Trust and Institute of Cancer Research, Fulham Road, London SW3 6JJ, United Kingdom; 2 Clinic for Tumors, Clinical Hospital Center, Rijeka 51000, Croatia

## Historical overview of the treatment for advanced gastrointestinal stromal tumors (GISTs)

GISTs represent a relatively rare entity; however, GISTs are still the most common mesenchymal neoplasm in the gastrointestinal tract. The majority of GISTs express the transmembrane receptor, KIT, a product of the *KIT* proto-oncogene that can lead to uncontrolled cell proliferation and resistance to apoptosis^1–3^. The prognosis of advanced GISTs at the beginning of the century was dismal. With chemotherapy response rates up to 5% and poor sensitivity to radiation, the median overall survival (OS) for GISTs was approximately 20 months^1^. The discovery of the tyrosine kinase inhibitor (TKI), imatinib, revolutionised the treatment of GISTs. Although imatinib was already in use for chronic myeloid leukemia and other Philadelphia chromosome–positive leukemias from the mid-1990s, prospective clinical trial data published in 2002 demonstrated the positive impact of imatinib in advanced GISTs. Whether the 400 or 600 mg dose, treatment with imatinib resulted in a remarkable 1-year OS of > 85%^2^, while an updated analysis showed a median progression-free survival (PFS) up to 2 years and an OS of 3.9 years with imatinib as first-line of treatment; however, the 10-year OS was 21.5% and the 10-year PFS was only 9.5%, highlighting the need for new treatment options in addition to imatinib^3^.

A flurry of positive trials in the ensuing 20 years led to the development of the treatment sequence (imatinib, sunitinib, regorafenib, and ripretinib), which is followed by most patients with advanced GISTs. In recent years we have witnessed significant breakthroughs in the molecular understanding of GISTs and circulating tumor DNA (ctDNA). Along with the discoveries of new effective TKI and non-TKI medications, those advances will most likely change the treatment landscape for advanced GISTs in the near future.

## Mutational profile as the basis for first-line treatment

While the first randomized trial with imatinib for advanced GIST treatment was published > 20 years ago, imatinib remains the first-line treatment of choice for the majority of patients; however, there is still an unmet need because patients with more uncommon types of GISTs, such as *KIT*/platelet-derived growth factor receptor alpha polypeptide (*PDGFRA*) wild-type [including succinate dehydrogenase (SDH)-deficient GIST], *KIT* exon 9, and *PDGFRA* D842V-mutated GIST, exhibit a significantly worse response to imatinib compared to patients with GIST harboring *KIT* exon 11 mutations. A change in the treatment paradigm in the first-line setting occurred in 2020 with publication of the NAVIGATOR trial. Despite being a phase I trial, unprecedented results were shown for avapritinib in patients with advanced *PDGFRA* D842V-mutated GIST. Avapritinib resulted in a clinical benefit rate of 98% (55/56 patients) and a PFS of 34.0 months. Although the median OS was not reached, OS at 36 months was 61% for any starting dose and 71% for patients with a 300/400 mg starting dose. Importantly, avapritinib was well-tolerated apart from neurocognitive toxicity. The most commonly reported adverse effects being nausea (69%), diarrhea (66%), and anemia (66%)^4,5^. The results of the NAVIGATOR trial, as well as of the tissue-agnostic trials that evaluated neurotrophic tropomyosin receptor kinase inhibitors (larotrectinib and entrectinib), and the case reports reporting the effectiveness of *Raf* murine sarcoma viral oncogene homolog B inhibitors in GISTs, have changed the clinical practice guidelines for the treatment of advanced GISTs^6^. Hence, treatment based on the mutational profile of GISTs is considered standard practice.

## Ripretinib and the value of ctDNA in second-line treatment

Similar to imatinib, sunitinib has remained the standard of care in the second-line treatment of advanced GISTs since 2006; however, the recent randomized phase III trial, INTRIGUE, demonstrated the value of ripretinib, a switch-control TKI active against a broad spectrum of *KIT* and *PDGFRA* mutations, in the same setting. An updated analysis of the INTRIGUE trial, which was published at ASCO in 2023, showed a similar PFS, OS, and response rate (RR) between ripretinib and sunitinib cohorts in the intention-to-treat (ITT) population. Furthermore, the PFS on next-line therapy was similar with both drugs, although a significant crossover was observed following study treatment. The majority of patients from the sunitinib arm received regorafenib (46.3%) or ripretinib (25.6%), while 61.5% of patients from the ripretinib arm received sunitinib followed by regorafenib (22.1%). Ripretinib demonstrated a significantly higher RR in the *KIT* exon 11 ITT (23.9% *vs.* 14.6%; *P* = 0.03) and less grade 3/4 adverse effects (42.6% *vs.* 67.4%), treatment interruptions (31.4% *vs.* 43.0%), and dose reductions (20.2% *vs.* 48.4%) compared to the sunitinib arm. The types of adverse effects were also different between the cohorts, with alopecia, fatigue, and myalgia reported as most common in the ripretinib arm and palmar-plantar erythrodysesthesia syndrome, diarrhea, and hypertension most common in the sunitinib arm^7,8^.

A particular value of the INTRIGUE trial lies in the additional analysis of ctDNA. The *post-hoc* analysis of the trial demonstrated a difference in the OS, PFS, and RR depending on the mutational profile. Patients with a *KIT* exon 11 and co-occurring *KIT* exon 17 and/or 18 mutations (mutations in the activation loop) derived greater benefit from ripretinib (HR = 0.22, 95% CI, 0.11–0.44, PFS: 14.2 *vs.* 1.5 months), while sunitinib was particularly effective in patients with *KIT* exon 11 and 13/14 mutations (mutations in the ATP-binding pocket) (HR = 3.94, 95% CI, 1.71–9.11, PFS: 4.0 *vs.* 15.0 months)^9^. These results led to the launch of the INSIGHT trial, the first clinical trial to select patients with GIST based on the ctDNA mutational profile^10^. The trial, which is set to be completed by 2027, could shed light on whether ctDNA could not only be used for obtaining often difficult-to-get tissue biopsies, but also to help choose treatment based on the type of mutation found in ctDNA.

## Recent clinical trials in the third-line setting

Recent trials have also challenged regorafenib, the standard of care in the third-line setting since 2013. Avapritinib, which became the new standard of care for advanced GISTs with a *PDGFRA* D842V mutation in the first-line setting since the NAVIGATOR trial^4,5^, was compared to regorafenib in the third-line setting in the VOYAGER trial. A similar PFS was obtained with both avapritinib and regorafenib in molecular-unselected GIST patients (4.2 *vs.* 5.6 months, HR = 1.25, 95% CI, 0.99–1.57; *P* = 0.055). The OS data were not mature at the time of publication, although 12-month OS estimates were 68.2% for avapritinib and 67.4% for regorafenib. Furthermore, avapritinib exhibited a higher RR (17.1% *vs.* 7.2%; *P* < 0.001). Notably, there were no significant differences in treatment-related adverse effects in this head-to-head trial. A similar number of patients experience grade 3/4 adverse effects in avapritinib (55.2%) and regorafenib (57.7%) arm. However, the types of adverse effects were different between the patients, with anemia (40.2%), nausea (39.3%), and fatigue (35.1%) being the most common in avapritinib arm, and plantar erythrodysesthesia syndrome (59.0%), diarrhea (34.6%), and fatigue (34.2%) more frequently reported in patients treated with regorafenib. Despite the trial having only 13 patients with GISTs harboring the *D842V* mutation, avapritinib was associated with a significantly longer PFS compared to regorafenib (*P* = 0.035)^11^. Avapritinib remains a valuable alternative to regorafenib even for patients without a *D842V* mutation, particularly when an objective response might be critical or adverse effects require a change of treatment.

## Recent clinical trials in the fourth-line setting

Ripretinib is currently under evaluation as an alternative to sunitinib as second-line treatment for advanced GISTs^7,8^. Ripretinib has become the standard of care as fourth-line treatment since the phase III trial, INVICTUS, was conducted in 12 countries and published in 2020. Ripretinib was associated with a longer PFS (6.3 *vs.* 1.0 months, HR = 0.15; *P* < 0.0001) and OS (15.1 *vs.* 6.6 months, HR = 0.36), and was effective even in patients with hard-to-treat wild-type GISTs compared to placebo. Furthermore, despite being tested in heavily pretreated patients, severe treatment-related adverse effects were only noted in 9% (8/85) of patients on ripretinib, most commonly an increase in lipase activity, hypertension, fatigue, and hypophosphatemia, compared to 7% (3/43) of patients who received placebo^12^. Only 4 of 85 (5%) patients in the ripretinib arm discontinued treatment. The value of ripretinib was further confirmed in a Chinese multicentre phase II trial, in which an even longer PFS (7.2 months) and a higher RR (18.4%) were achieved compared to the original trial. The trial also confirmed the excellent tolerability of ripretinib; only 15.4% of the patients exhibited grade 3/4 adverse effects^13^.

The discovery that cytoplasmic heat shock protein (HSP) is required for the folding and stabilization of KIT and PDGFRA, and that a selective HSP90 inhibitor resulted in apoptosis and growth inhibition of tumor cells gave rise to the CHAPTER-GIST-301 trial. The trial, which was conducted in Japan and published in 2022, demonstrated a longer PFS (2.8 *vs.* 1.4 months, HR = 0.51; *P* = 0.006) and OS (13.8 *vs.* 7.6 months, HR = 0.42; *P* = 0.007) for the HSP90 inhibitor, pimitespib, compared to placebo. The pimitespib arm reported more grade 3/4 adverse effects [43.1% *vs.* 28.6% (25 *vs.* 8 patients)] than the placebo arm, most commonly diarrhea (13.8%, *n* = 8), anemia, and decreased appetite (both 6.9%, *n* = 4). Although pimitespib was relatively well-tolerated, 8 patients (13.8%) developed night blindness; no patients in the placebo arm developed night blindness^14^. Although pimitespib adds another treatment option in the fourth-line treatment of advanced GISTs, no studies comparing pimitespib to ripretinib are available.

## Role of immunotherapy and chemotherapy in the treatment of advanced GISTs

While TKIs remain the mainstay of treating advanced GIST, the CHAPTER GIST-301 trial demonstrated the value and potential of non-TKI medications. A non-comparative, unblinded phase II trial evaluated the efficacy of checkpoint inhibitors, anti-programmed death 1 monoclonal antibody (nivolumab) with or without anti-cytotoxic T lymphocyte antigen-4 inhibitor (ipilimumab), in the second-line treatment of metastatic GISTs. The use of checkpoint inhibitors, which primarily act by blocking the interaction between T-cells and the tumor, facilitate attack of the cancer by the immune cells, resulted in a clinical benefit rate of 52.6% in advanced GISTs, with one patient achieving a complete response^15^.

Another emerging non-TKI treatment option is the oral alkylating agent, temozolomide. Although chemotherapy is generally considered ineffective in advanced GISTs, in 2022 Yebra et al.^16^ demonstrated that temozolomide achieved a disease control rate of 100% with a median OS of 1.9 years from the start of treatment in 5 patients with SDH-deficient GISTs. The importance of this finding lies in the fact that SDH-deficient GISTs are commonly found in younger patients, often metastasize, and are considered resistant to the usual first-line treatment with imatinib^16^. Although the immunotherapy trial did not meet the primary endpoint (> 15% RR), and the temozolomide study only included 5 patients in the clinical setting, Yebra et al.^16^ demonstrated the potential of different drug classes besides TKIs, which will only broaden the spectrum of treatment options against advanced GISTs in select patients. Future research is critical to adequately position these mediations in the existing treatment scheme.

## The future of systemic treatment in the management of advanced GISTs

In addition, there are a number of very promising agents in development. In early 2023, a small molecule imidazopyridine (IDRX-42) demonstrated excellent kinase selectivity and a relative shrinkage of tumors (up to 57.3%) in patient- and cell line–derived xenograft models^17^. The results of the clinical trial not only with IDRX-42 but also of NB003 in advanced GIST are also eagerly awaited^18,19^. Finally, along with monotherapy, one possible way forward is to combine therapies, which is the goal of the phase III PEAK trial conducted in the second-line setting. The trial will test the combination of sunitinib and a selective oral TKI (bezuclastinib) against monotherapy and is expected to be completed by 2026^20^ (**Table 1**).

**Table 1:** Brief overview of some of the most awaited interventional clinical trials in advanced GISTs

Name of the trial	Phase	Tested medications	Setting	Expected completion date	Primary outcome measures
INSIGHT^10^ (NCT05734105)	III	Ripretinib *vs.* sunitinib	Second-line, co-occurring KIT exons 11+17/18 mutations are without KIT exon 9, 13, or 14 mutations	December 2027	PFS
PEAK^20^ (NCT05208047)	III	CGT9486 plus sunitinib *vs.* sunitinib	Second-line	September 2026	PFS, C_max_, T_max_, AUC
NCT04936178^19^	I	NB003	Second-line or beyond. Other cancers with KIT or PDGFRα gene alterations were also included.	December 2025	Incidence of dose-limiting toxicities and adverse effects, ORR, and DOR
NCT05160168^18^	I/II	THE-630	Third-line or beyond	February 2024	DLT; RP2D; MTD; ORR
NCT05489237^21^	I/Ib	IDRX-42	Second-line or beyond	September 2026	DLT; RP2D; MTD; ORR; TEAE

AUC, area under the plasma concentration-time curve; C_max_, maximum plasma concentration; DLT, dose-limiting toxicity; DOR, duration of response; GIST, gastrointestinal stromal tumor; KIT, c-kit proto-oncogene product; MTD, maximum tolerated dose; ORR, objective response rate; PDGFRα, platelet-derived growth factor receptor A; PFS, progression-free survival; RP2D, recommended phase 2 dose; T_max_, time to maximum observed plasma concentration; TEAE, treatment emergent adverse events.

While GIST is a rare disease, there is a plethora of different TKI and non-TKI medications with proven efficacy^6^, and additional new drugs are under development. As the understanding of molecular mechanisms deepens, greater precision in the management of GISTs will undoubtedly occur. The results of the trials in which treatment selection is based on the ctDNA mutational profile are eagerly awaited and could shape how we approach the management of GISTs in the near future. Furthermore, having more head-to-head trials with similar efficacy, but a different profile of adverse effects and effect on the quality of life, allows for a more personalised approach for each patient with advanced GISTs. While the future treatment of metastatic GIST is bright, the growing knowledge in the field further emphasises the need to treat GIST patients in a multidisciplinary team setting with experience in treating sarcomas.
